# Echocardiography-Based Pulmonary Artery Pulsatility Index Correlates with Outcomes in Patients with Acute Pulmonary Embolism

**DOI:** 10.3390/jcm14082685

**Published:** 2025-04-14

**Authors:** Gassan Moady, Loai Mobarki, Tsafrir Or, Alexander Shturman, Shaul Atar

**Affiliations:** 1 Department of Cardiology, Galilee Medical Center, Nahariya 2210001, Israel; mobarki.loai@hotmail.com (L.M.); tsafriro@gmc.gov.il (T.O.); alexanders@gmc.gov.il (A.S.); shaula@gmc.gov.il (S.A.); 2 Azrieli Faculty of Medicine, Bar Ilan University, Safed 2210001, Israel

**Keywords:** pulmonary embolism, pulmonary artery pulsatility index, right ventricle, mortality

## Abstract

**Objectives**: The pulmonary artery pulsatility index (PAPI) is a novel hemodynamic parameter that reflects right ventricular (RV) function. PAPI was shown to be useful in predicting outcomes following left ventricular assist device (LVAD) implantation, acute RV infarction, and in patients with chronic RV failure. The standard method to estimate PAPI is during right heart catheterization (RHC); however, echocardiography-based PAPI was also shown to be accurate. In the current study, we evaluated the ability of echocardiography-based PAPI to predict outcomes of patients with acute pulmonary embolism (PE). **Methods**: A total of 177 patients (mean age 67 ± 15, 54.1% male) with acute PE were included in the study. PAPI was calculated based on measurements from standard transthoracic echocardiography. **Results**: 27% of patients needed oxygen support, 5.6% were on mechanical ventilation, and 7.3% were on inotropic support. The 30-day mortality rate in the whole cohort was 8.3%. Lower PAPI measurements were associated with increased 30-day mortality (*p* < 0.05), a higher rate of RV failure (*p* < 0.001), and the need for inotropic support (*p* < 0.05). There was no association between PAPI and the need for oxygen support (*p* = 0.59), mechanical ventilation (0.06), or length of stay (LOS) (*p* = 0.414). PAPI was superior to tricuspid annular plane systolic excursion (TAPSE) in predicting mortality and RV failure. **Conclusions:** Echocardiography-derived PAPI is feasible and superior over TAPSE in predicting RV failure and mortality among patients with acute PE.

## 1. Introduction

The pulmonary artery pulsatility index (PAPI) is a novel hemodynamic parameter derived from the ratio of pulmonary artery pulse pressure to right atrial pressure (RAP) and reflects right ventricular (RV) function [1]. The standard method to calculate PAPI is during right heart catheterization (RHC) based on the following formula: PAPI= (PASP-PADP)/RAP, where PASP and PADP are the pulmonary artery systolic and diastolic pressures, respectively [2,3]. PASP is estimated from tricuspid regurgitation velocity whereas PADP can be calculated from pulmonary regurgitation velocity, and RAP might be estimated via inferior vena cava diameter and collapsibility [4]. In previous studies, echocardiographic estimation of PAPI was shown to be applicable and accurate [5,6,7]. In their retrospective study, Mirza et al. evaluated 20 patients who had undergone RHC and transthoracic echocardiography within a two-week timeframe, and they found a positive correlation between echocardiographic and invasive PAPI with improved correlation in lower PAPI values [7]. The clinical significance of PAPI has been demonstrated in several diseases that affect RV function and has been in use for decision-making in such cases. In patients with advanced HF, lower PAPI is associated with increased mortality and has a prognostic value in patients scheduled for heart transplantation or left ventricular assist device (LVAD) implantation [8,9,10,11]. In cardiogenic shock, PAPI < 1.0 at 2 h after diagnosis and therapy establishment was associated with increased 30-day mortality [12]. Likewise, in patients with acute inferior myocardial infarction, PAPI provides high sensitivity and specificity in predicting in-hospital mortality [13]. Moreover, PAPI was shown to predict cardiovascular outcomes and hospitalizations for heart failure across a hospital-based sample regardless of the background disease [14]. Acute pulmonary embolism (PE) is a common cardiovascular disease that is associated with high morbidity and mortality rates [15]. Risk stratification of patients with PE is based on clinical, laboratory, and echocardiographic parameters [16,17]. The Pulmonary Embolism Scoring Index (PESI) is commonly used for this purpose and it is also available in its simplified version [17]. Hemodynamic changes in acute PE are mainly determined by the degree of RV involvement that is typically evaluated during standard echocardiography using visual estimation of RV function, RV diameter and volume, and tricuspid annular plane systolic excursion (TAPSE) [18]. While TAPSE is an easily obtained surrogate marker, it is load-dependent and does not always reflect the global RV function [19]. The use of PAPI for risk stratification in patients with acute PE has not been evaluated yet. In the current study, we evaluated the association between echocardiography-derived PAPI and clinical outcomes among patients hospitalized for acute PE in a retrospective cohort.

## 2. Methods

### 2.1. Study Population

**Figure 1:**
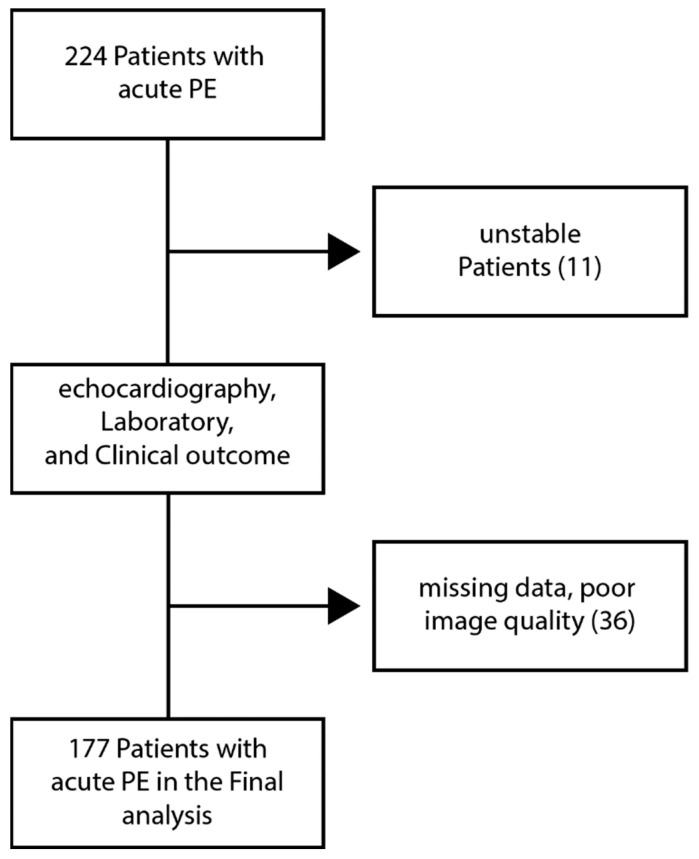
Study flowchart. A total of 242 patients with discharge diagnoses of “acute pulmonary embolism” were identified between 2018 to 2022. We excluded 33 patients due to missing data and 11 patients due to hemodynamic instability on admission. Finally, 177 patients were included in the analysis.

The study included patients hospitalized for acute PE between the years 2019 and 2022 in cardiology, intensive cardiac care, and intensive care units. The diagnosis of acute PE was confirmed by computed tomography angiography in most cases while perfusion imaging was performed in cases of severe iodine allergy or advanced renal failure. Patients defined as “high probability” for PE according to perfusion imaging were considered as having acute PE. All patients were treated according to the guidelines of acute PE. Hemodynamic parameters and need for oxygen support were assessed on presentation whereas laboratory and echocardiographic parameters were obtained during the first two days upon admission. All echocardiographic parameters were analyzed by a cardiologist specialized in echocardiography. The simplified pulmonary embolism severity index (SPESI) upon admission was used to dictate treatment, in which age >80 years, history of cancer, history of cardiopulmonary disease, systolic blood pressure <90 mmHg on presentation, heart rate >110 beats/minute, and oxygen saturation <90% give one point each. Low-risk and intermediate-low-risk patients were treated with oral anticoagulation while high-risk patients or patients with intermediate-high risk with deterioration were treated with intravenous recombinant tissue plasminogen activator (rTPA), 100 mg, over 2 h. In patients with background anticoagulation therapy, the dosage of oral anticoagulation was increased according to the recommended guidelines of PE treatment. Mechanical ventilation and intravenous inotropes (norepinephrine +/− dobutamine) were used as indicated for respiratory and hemodynamic support, respectively. In selected cases, endovascular ultrasound-facilitated interventions were performed to dissolve large thrombus. Patients with missing laboratory or echocardiographic data were excluded from the study. The study flowchart is summarized in Figure 1.

### 2.2. Echocardiographic Measurements

**Figure 2:**
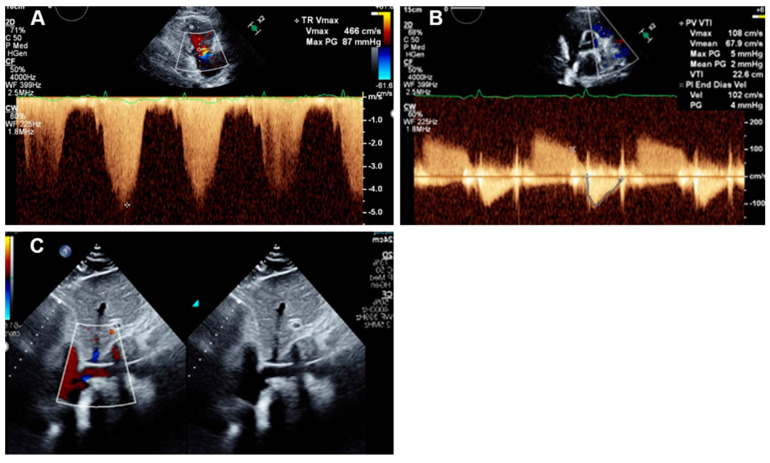
Illustrative images for echocardiography estimation of PAPI. (**A**) PASP estimation based on TR signal using the formula PASP = 4 (tricuspid valve regurgitation velocity) ^2^ +RAP. (**B**) PADP estimation PADP = 4 (end-diastolic pulmonary regurgitation velocity) ^2^ +RAP. (**C**) RAP estimation based on IVC collapsibility during inspiration.

All echocardiographic measurements were performed using a Philips Epiq-7 machine and Epiq X8-2t transducer (Philips, Adnover, MA, USA). Data were analyzed by the same cardiologist, who specialized in echocardiography. Briefly, RAP was estimated based on vena cave diameter and collapsibility. A normal-sized inferior vena cava (<2.0 cm) and normal respiratory variation with inspiration (>50% decrease) indicate normal right atrial pressure (<10 mm Hg). A mildly increased diameter (2.0 to 2.5 cm) or decreased respiratory variation signifies mildly elevated RAP (10 to 15 mm Hg), and a dilated inferior vena cava (>2.5 cm) with decreased respiratory variation signifies highly elevated RAP (>15 mm Hg). PASP was calculated based on peak tricuspid valve regurgitation signal and estimated RAP using the formula PASP = 4 (tricuspid valve regurgitation velocity)^2^ +RAP [4]. PADP was calculated based on pulmonic valve regurgitation signal as PADP = 4 (end-diastolic pulmonary regurgitation velocity)^2^ +RAP [4]. Continuous-wave Doppler of the tricuspid regurgitation jet was carried out in the RV inflow, short axis, and apical four-chamber view. The highest velocity obtained was used to calculate PASP. TAPSE was determined in M-mode echocardiography by aligning the lateral tricuspid annulus with the ventricular apex in the apical four-chamber view and measuring the lateral annular displacement [4]. Illustrative images of echocardiographic measurements are depicted in Figure 2.

### 2.3. Laboratory Data

Maximal values of troponin-I and N-terminal pro-brain natriuretic peptide (NT-proBNP) were collected for each patient during the index hospitalization. High sensitivity troponin I level was measured using ARCHITECT c-TnI assay, Abbott. Cut-off values for abnormal troponin levels were above 20 ng/L and 30 ng/L for men and women, respectively. NT-proBNP is expressed in pg/mL and was determined using electrochemi-luminescence Elecsys immunoassay (Abbott Laboratories, Dundee, UK)), in which abnormal levels are above 125 pg/mL.

### 2.4. Outcomes

The outcomes included in-hospital and 30-day mortality, need for cardiopulmonary resuscitation, RV failure, oxygen support, mechanical ventilation, thrombolytic therapy, and inotropic support during the index hospitalization. RV failure was defined as signs of peripheral volume overload (leg edema or jugular venous distention) requiring intravenous diuretics.

### 2.5. Statistical Analysis

Data are presented as mean with a standard of deviation (SD) or median with interquartile range (IQR) for continuous variables and percentages for categorical variables. Association between continuous variables with normal distribution was performed using Spearman’s P test. Comparison between continuous variables was performed with an independent sample t-test or Mann–Whitney test. Comparison between percentages was performed using a Chi-square or Fischer exact test (when expectancy < 5). We used multiple logistic regression for 30-day mortality prediction. An alpha value < 0.05 was considered statistically significant. The receiver operating characteristic (ROC) curve was used to demonstrate the performance of PAPI in predicting 30-day mortality. All statistical analysis was performed using IBM SPSS statistics, version 27.

The study was conducted according to the guidelines of the Declaration of Helsinki and approved by the local ethical committee of Galilee Medical Center, Nahariya, Israel.

## 3. Results

A total of 177 patients (mean age 67, 54% male) were included in the final analysis. Overall, 33% of them had ischemic heart disease and about 15% had heart failure. In about 11%, background anti-coagulation was documented (68% with apixaban). The median troponin level was 21 ng/L [IQR, 6–138]. The baseline characteristics and laboratory data of the study population are summarized in Table 1.

The mean LVEF was 56%, and all patients were hemodynamically stable upon admission without respiratory or inotropic support. While echocardiographic RV dysfunction (TAPSE < 17) was documented in 25% of the patients, clinical RV failure (signs of peripheral edema and jugular congestion mandating diuretic therapy) was observed in about 18%. Inotropic support (in about 6%) of patients and mechanical ventilation (in about 7%) were established based on hemodynamic parameters and clinical judgment. The echocardiographic and hemodynamic parameters are summarized in Table 2.

We divided patients into two risk scores, low risk vs. intermediate-low/intermediate-high risk. The baseline characteristics of the two groups are given in Table 3.

### 3.1. Outcomes

Of the whole cohort, 15 (8.5%) patients died within 30 days from the index hospitalization; all of them had clinical and echocardiographic signs of heart failure. Patients who died had lower PAPI values (1.9 ± 1.5 vs. 4.9 ± 2.4, *p* < 0.05). Lower PAPI was also associated with a higher rate of RV failure (*p* < 0.05) and the need for inotropic support (*p* < 0.05). There was no association between PAPI and LOS (*p* = 0.41), need for oxygen (*p* = 0.59), or need for mechanical ventilation (0.06). After multivariate logistic regression, low TAPSE, high SPESI, and low PAPI were associated with increased 30-day mortality. The association of different parameters with 30-day mortality is given in Table 4.

**Figure 3:**
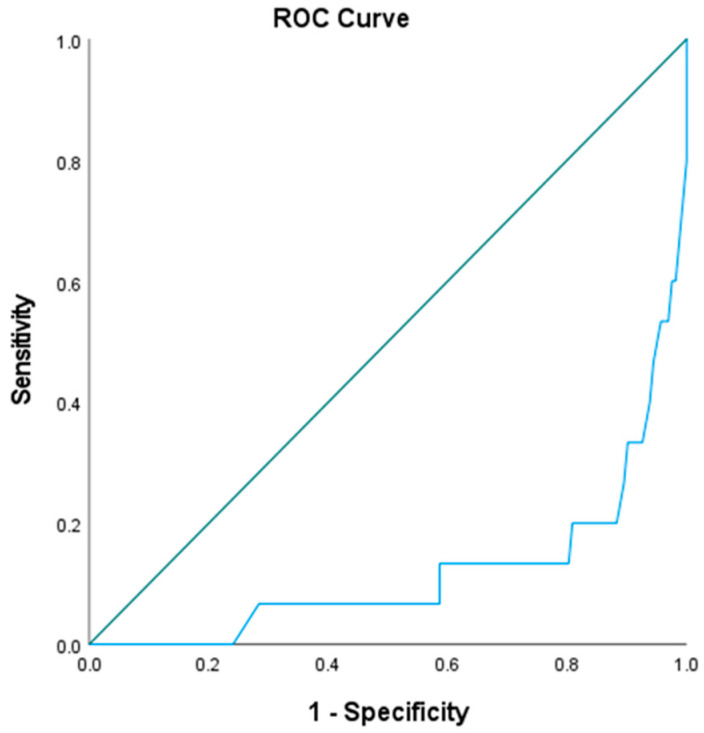
ROC curve of PAPI for predicting 30-day mortality.

When determining a cut-off lower value of 1.6, PAPI has a strong correlation with 30-day mortality [OR = 19.6, 95% CI (8–66), *p* < 0.05]. The ROC curve of PAPI in predicting outcomes when the cut-off lower value is 1.6 is illustrated in Figure 3.

**Figure 4:**
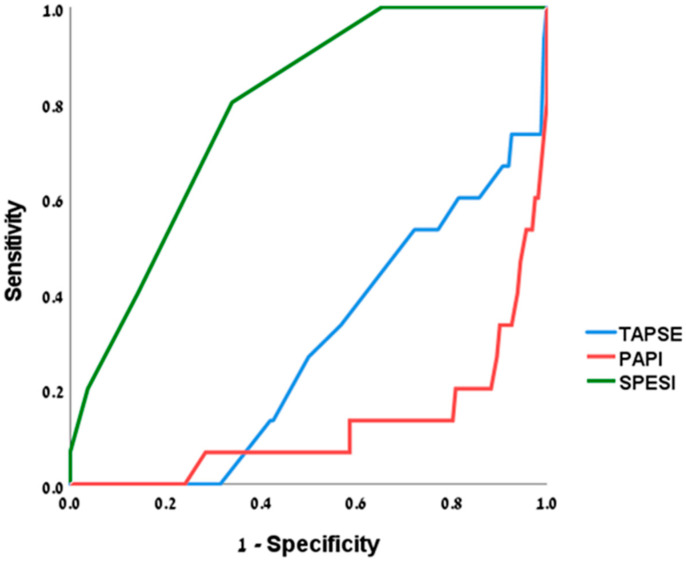
PAPI performance compared to TAPSE and SPESI. ROC curves for TAPSE (blue line), PAPI (red line), and SPESI (green line) show the performance of each parameter in predicting 30-day mortality.

The ROC curves for PAPI, TAPSE, and SPESI in PE are given in Figure 4.

### 3.2. Discussion

The PAPI is a novel hemodynamic parameter of RV function that has been validated in several acute and chronic conditions. PAPI is calculated based on PASP, which is an indirect indicator of RV contractility against afterload, PADP that reflects left ventricular filling pressure, and RAP, which in high values, indicates a failing RV [14]. The interaction between RAP, wedge pressure, pulmonary artery capacitance, and stroke volume ultimately determines the value of PAPI [9,18,19,20]. In the current study, we showed a strong correlation between echocardiographic PAPI and outcomes in patients with acute PE. The overall 30-day mortality rate in our cohort (hemodynamically stable patients) was 8.5%. The mortality rate in acute PE varies significantly depending on background diseases, age, severity of the embolism, and local expertise in invasive therapy in the appropriate cases. Overall, a decreasing trend in mortality is observed [21], probably due to improvements in treatment strategies and approach algorithms led by multidisciplinary teams. In the current study, we excluded unstable patients and included only low-risk, intermediate-low-risk, and intermediate-high-risk patients. The cause of death was reported as “respiratory failure” in about 40% and “cardiac arrest” or “cardiopulmonary arrest” in about 60%. Since RV dysfunction is a key parameter in PE hemodynamics, we assumed that PAPI might be a good predictor for mortality in both acute PE and chronic thromboembolic pulmonary hypertension. In the setting of acute PE, elevation in pulmonary artery resistance leads to sustained RV pressure and hemodynamic changes that correlate with clinical outcomes. Invasive measurement during RHC is considered the gold standard for PAPI evaluation; however, echocardiography-derived PAPI was shown to be feasible and accurate for risk stratification in patients with pulmonary hypertension and in patients with HFrEF [5,6]. Since routine RHC in hemodynamically stable patients with acute PE is not justified, we used echocardiography-based PAPI. Evaluation of RV function in acute PE by transthoracic echocardiography is mainly performed by eyeballing and TAPSE measurement. Several echocardiographic parameters have been suggested for risk stratification in acute hemodynamically stable PE including RV diameter, the presence of McConnell’s sign (RV free wall akinesis with apical sparing), pulmonary artery pressure, and TAPSE [22,23]. Among these parameters, the TAPSE/PASP index is considered a strong predictor of RV function since it incorporates changes in RV function and pulmonary afterload and has been validated in heart failure, pulmonary arterial hypertension, and also in acute PE [24,25,26,27]. In acute intermediate-risk PE, TAPSE/PASP was superior to TAPSE or PADP alone in predicting 30-day mortality, and a cut-off value of 0.4 was determined for this purpose [24]. Currently, the combination of clinical scores such as PESI and SPESI scores with echocardiographic parameters is considered the optimal way for risk stratification in PE [28]. We found a correlation between low PAPI and inotropic support but not with oxygen use or mechanical ventilation. Since we included only hemodynamically stable patients on presentation, our results indicate a potential ability of PAPI to predict hemodynamic deterioration. The need for inotropic support in PE is mainly determined by the degree of RV dysfunction while hypoxia is mediated by the amount of ventilation–perfusion mismatch and dead space ventilation that hamper respiratory function, depending on the anatomy and size of thrombi [29]. The rapid increase in pulmonary vascular resistance mediated by local and neurohormonal mechanisms leads to an increase in RV tension wall, flattening and left displacement of the interventricular septum, and reduction in left ventricular distensibility and filling during diastole [30]. Moreover, elevated RV filling pressure reduces blood supply and increases oxygen demand, which further jeopardizes RV contractility. If calculated promptly in combination with clinical scores, PAPI may be a strong predictor of hemodynamic deterioration in acute PE. The incorporation of the simple echocardiographic PAPI along with clinical scores such as SPESI may have an additive value for risk assessment and prediction of hemodynamic deterioration of stable patients. Patients with low PAPI upon presentation may benefit from intensive care management and intravenous heparin therapy rather than oral anticoagulation due to the risk of rapid deterioration and the potential need for circulatory support or invasive thrombectomy techniques. The lack of association between PAPI and LOS in our study is not surprising since LOS depends on several factors and may not be predictable by a single parameter [31]. Moreover, patients with lower PAPI had higher mortality rates and this may also be reflected by shorter LOS. Although different thresholds have been suggested as cut-off values of PAPI in clinical studies, no specific value has been determined. Nevertheless, it is undisputed that the lower the PAPI, the higher the risk for RV failure and cardiovascular events. The range of values for PAPI may be very wide depending on the study population. In their large cross-sectional study, Zern et al. found that patients in the lowest PAPI quartile had a greater risk of mortality, HF hospitalization, and cardiovascular events even at values higher than previously reported [14]. In one study, a cut-off lower value of 3.9 of PAPI was independently associated with lower survival rates in patients with inoperable chronic thromboembolic pulmonary hypertension [32]. Currently, PAPI is considered a strong predictor of clinical outcomes in several chronic conditions such as advanced HF patients scheduled for LVAD or heart transplantation [3,8,10]. Based on our results and the current physiological and clinical data, we assume that PAPI may be used for risk stratification and predicting hemodynamic deterioration in acute PE. Larger studies are warranted to identify a “high risk” range of PAPI values that identify patients at risk of hemodynamic deterioration in PE.

### 3.3. Limitations

Our study has several limitations. First, owing to the retrospective design of the study, it might be difficult to account for all potential confounders. Second, estimation of PASP and PADP depends on accurate measurement of tricuspid and pulmonic regurgitation, respectively, and this may not be applicable in all patients depending on the quality of images. RAP is also indirect and it is estimated based on vena cava collapsibility and variation with inspiration. However, echocardiographic assessment is still preferred and easily obtained in the majority of stable patients to achieve these parameters rather than the invasive way. Third, we excluded hemodynamically unstable patients and those treated with invasive thrombectomy techniques so the results are not applicable for these patients. Unstable patients with hypotension are usually treated with urgent thrombolytic therapy in order to relieve pressure on the failing RV. We do not think that detailed echocardiographic assessment is applicable to these patients. Furthermore, the measurement of pulmonary artery pressure in unstable patients with severe hypotension may be not accurate and does not reflect the severity of the disease. Unstable patients are usually treated with urgent intravenous thrombolysis and mechanical support when needed, regardless of any echocardiographic parameter.

## 4. Conclusions

Echocardiographic PAPI can be easily assessed during standard echocardiography. Our findings suggest that PAPI may predict short-term outcomes and hemodynamic deterioration in the setting of acute hemodynamically stable PE.

## Figures and Tables

**Table 1:** Baseline characteristics of the study population.

n	177
Age (mean ± SD), years	67 ± 15
Male (%)	96 (54.2)
BMI (mean ± SD), Kg/m^2^	23.2 ± 3.1
Hypertension (%)	122 (68.9)
Hyperlipidemia (%)	103 (58.2)
Diabetes mellitus (%)	62 (35)
Chronic kidney disease (%)	38 (21.5)
Tobacco use (%)	109 (61.9)
Ischemic heart disease (%)	56 (31.6)
History of stroke (%)	14 (7.9)
Heart failure	26 (14.7)
Pacemaker/ICD (%)	22 (12.4)
Medications	
ACE inhibitor/ARB (%)	85 (48)
Beta blockers (%)	105 (59.3)
Aspirin (%)	92 (52)
Statins (%)	122 (68.9)
Spironolactone (%)	31 (17.5)
SGLT2 inhibitors (%)	70 (39.5)
Anticoagulants (%)	19 (10.7)
Hemoglobin (mean ± SD), gr/dL	12.7 ± 2.3
WBC (mean ± SD), × 10^9^/L	10.4 ± 3.9
Creatinine (mean ± SD), mg/dL	1.1 ± 0.6
CRP (median, IQR), mg/L	56 [23, 154]
Troponin (median, IQR), ng/L	21 [6, 138]

ACE, angiotensin converting enzyme; ARB, angiotensin receptor blocker; BMI, body mass index; CRP, C-reactive protein; ICD, implantable cardiac defibrillator; SGLT2, Sodium-glucose co-transporter; WBC, white blood cells.

**Table 2:** Echocardiographic and hemodynamic measurements.

n	177
SBP (mean ± SD), mmHg	131 ± 22
Hear rate (mean ± SD), bpm	87 ± 17
LVEF (mean ± SD), (%)	56 ± 11
Estimated RAP (median, IQR), mmHg	6 [5, 10]
Estimated PASP (median, IQR), mmHg	40 [30, 55]
Estimated PADP (median, IQR), mmHg	10 [9, 15]
Estimated PAPI (median, IQR)	4.3 [2.9, 6]
TAPSE (mean ± SD), cm	1.9 ± 0.5
<1.7 (%)	44 (25)
>1.7 (%)	133 (75)
SPESI score (median, IQR)	1 [0, 2]
RV involvement (%)	33 (18.6)
Need for oxygen (%)	47 (27)
Mechanical ventilation (%)	13 (7.3)
Inotropes (%)	10 (5.6)
Thrombolysis (%)	8 (4.5)
CPR (%)	15 (8.5)
Length of stay (median, IQR), days	6 [4, 10]
30-day mortality (%)	15 (8.5)

CPR, Cardiopulmonary resuscitation; LVEF, left ventricular ejection fraction; PADP, pulmonary artery diastolic pressure; PAPI, pulmonary artery pulsatility index; PASP, pulmonary artery systolic pressure; RAP, right atrial pressure; RV, right ventricle; SBP, systolic blood pressure; SPESI, simplified pulmonary embolism severity index; TAPSE, tricuspid annular plane systolic excursion.

**Table 3:** Baseline characteristics of patients stratified by risk (low vs. intermediate-low/intermediate-high).

	Low Risk	Intermediate-High Risk	*p* Value
n	111	66	
Age (mean ± SD), years	65 ± 18	70 ± 10	<0.05
Male (%)	58 (52%)	38 (57%)	0.49
BMI (mean ± SD), Kg/m^2^	22.1 ± 2.5	25 ± 3.1	<0.05
Diabetes mellitus (%)	37 (33)	25 (38)	0.54
Hypertension (%)	76 (69)	46 (70)	0.86
Hyperlipidemia (%)	67 (60)	36 (55)	0.45
Tobacco use (%)	70 (63)	39 (59)	0.59
Chronic kidney disease (%)	23 (21)	15 (23)	0.75
Ischemic heart disease (%)	38 (34)	18 (27)	0.34
History of stroke (%)	7 (6)	7 (11)	0.31
Pacemaker/ICD (%)	16 (14)	6 (9)	0.29
Hemoglobin	12.6 ± 2.1	12.9 ±2.5	0.39
LVEF (%)	56 ± 12	55 ± 8	0.54

**Table 4:** Multivariate logistic regression for 30-day mortality.

	OR	95% CI	*p* Value
Age	1.03	0.96–1.09	0.48
Male	1.5	0.28–8.3	0.63
Diabetes mellitus	5.3	0.76–37.0	0.09
Chronic kidney disease	3.1	0.87–11.2	0.08
TAPSE	0.09	0.0001–0.186	<0.05
SPESI	3.2	1.5–6.6	<0.05
Low PAPI	0.14	0.045–0.43	<0.05

CI, confidence interval, OR, odds ratio, PAPI, pulmonary artery pulsatility index, SPESI, simplified pulmonary embolism severity index; TAPSE, tricuspid annular plane systolic excursion.

## Data Availability

The data presented in the study are available upon request from the corresponding author, G.M.
